# Hypoxia/Aglycemia-Induced Endothelial Barrier Dysfunction and Tight Junction Protein Downregulation Can Be Ameliorated by Citicoline

**DOI:** 10.1371/journal.pone.0082604

**Published:** 2013-12-16

**Authors:** Xiaotang Ma, Huiting Zhang, Qunwen Pan, Yuhui Zhao, Ji Chen, Bin Zhao, Yanfang Chen

**Affiliations:** 1 Guangdong Key Laboratory of Age-Related Cardiac and Cerebral Diseases, Institute of Neurology, Affiliated Hospital of Guangdong Medical College, Zhanjiang, China; 2 Department of Pharmacology & Toxicology, Boonshoft School of Medicine, Wright State University, Dayton, Ohio, United States of America; Georgia Regents University, United States of America

## Abstract

This study explores the effect of citicoline on the permeability and expression of tight junction proteins (TJPs) in endothelial cells under hypoxia/aglycemia conditions. Hypoxia or oxygen and glucose deprivation (OGD) was utilized to induce endothelial barrier breakdown model on human umbilical vein endothelial cells (HUVECs) and mouse brain microvascular endothelial cells (bEnd.3s). The effect of citicoline on endothelial barrier breakdown models was determined at either low or high concentrations. FITC-Dextran flux was used to examine the endothelial permeability. The expression of TJPs was measured by immunofluorescence, Real-time PCR and Western Blot methods. Results showed that hypoxia or OGD increased the permeability of HUVECs accompanied with down-regulation of occludens-1 (ZO-1) and occludin at both mRNA and protein levels. Similarly in bEnd.3s, hypoxia increased the permeability and decreased the expression of ZO-1 and claudin-5. Citicoline treatment dose-dependently decreased the permeability in these two models, which paralleled with elevated expression of TJPs. The data demonstrate that citicoline restores the barrier function of endothelial cells compromised by hypoxia/aglycemia probably via up-regulating the expression of TJPs.

## Introduction

Citicoline (CDP-choline, cytidine diphosphate choline, cytidine 5′-diphosphocholine) is composed of ribose, pyrophosphate, cytosine and choline; clinically, CDP-choline is commonly used for treating various types of neurodegenerative diseases, such as Amyotrophic lateral sclerosis (ALS), Multiple sclerosis (MS) and Alzheimer disease (AD) [1]. More recent evidences suggest that citicoline may increase dopamine production and glutamate uptake in the brain to improve cognition [2], [3]. Citicoline could also reduce free fatty acid release and recover the activities of mitochondrial ATPase and membrane Na^+^/K^+^ ATPase to alleviate brain damage [4], [5]. However, the pathophysiology of neurodegenerative diseases is complex which includes cholinergic deficit, glutamate excitatory [6], neuroinflammation [7], immunity dysregulation [8], glucose hypometabolism and blood–brain barrier (BBB) disruption [9], [10]; therefore the underlying mechanisms of citicoline's therapeutic effects on neurological diseases are largely unknown.

Endothelial cells play an important role in BBB function; BBB dysfunction is recognized to participate in neurodegenerative disorders [11], [12]. Cerebral vascular endothelial cells develop highly selective barrier which controls the exchanges between blood and brain compartments for the maintenance and regulation of the neuronal microenvironment [13]. Tight junctions proteins (TJPs) such as zonula occludens-1 (ZO-1), occludin and the claudin family exist in cerebral vascular endothelial cells, which are the most crucial factors modulating barrier integrity [14] [15]. It has been suggested that BBB dysfunction in AD is likely related to the injury or dysregulation of TJPs [16].

In the study described herein, we examine the therapeutic effect of citicoline on hypoxia/aglycemia-induced endothelial barrier breakdown as well as the correlated changes in TJP expression.

## Materials and Methods

### Cell Culture

Human umbilical vein endothelial cells (HUVECs) and mouse brain microvascular endothelial cells (bEnd.3s) were obtained from Shanghai Bioleaf Biotech Co., Ltd. The cells were cultured on poly-D-lysine-coated six-well plates at a density of 1×10^5^ cells/well in DMEM and DMEM/F12 (Invitrogen, Carlsbad, CA, US), respectively, supplemented with 10% fetal bovine serum (FBS; GBICO), 100 U/ml penicillin and 100 U/ml streptomycin in a 37°C incubator with humidified atmosphere of 5%CO_2_/95% air. The media were changed every 2 days. After reaching confluence, cells were passaged using 0.1% trypsin-EDTA (GIBCO, Grand Island, NY, USA). The 4^th^ passage of cells was used in this study.

### Endothelial Barrier Breakdown Models and Citicoline Treatments

After reaching confluent, cultured HUVECs and bEnd.3s were used to build endothelial barrier breakdown models. In the hypoxia model, HUVECs and bEnd.3s were exposed to hypoxic (1% O2) condition for 24h using a hypoxic chamber as previously described [17]. In the oxygen and glucose deprivation (OGD) model, HUVECs were cultured in glucose-free DMEM medium and hypoxic (1% O2) condition for 6 h [18]. OGD was not produced in bEnd.3s. For evaluating the treatment effect of citicoline on these models, citicoline (pharmaceutical factory affiliated to Guangdong Medical College, Zhan Jiang, China) was applied to cells during the period of hypoxia or OGD at low dose (0.1 mmol/L) or high dose (1 mmol/L) [19], [20].

### Real-time RT-PCR Analysis

Total RNA was extracted with TRIzol reagent (Invitrogen, Carlsbad, CA, USA). cDNA was synthesized using RevertAid First Strand cDNA Synthesis Kit (Thermo Scientific) according to the manufacturer's protocol. Real-time PCR was carried out on a LightCycer480-II System (Roche Diagnostics, Penzberg, Germany) using SYBR® Premix Ex TaqTM (TAKARA). Gene-specific oligonucleotides were obtained from sangon (QuantiTect Primer Assay). PCR primers were: 5- GCA CAT GCA GTG CAA GGT GTA TGA-3 and 5- AAG GTA ACA AAG AGT GCC ACC AGC-3 for claudin-5; 5-TAC AGC AAT GGA AAA CCA CAC T-3 and 5-CAA AGG AAT GGG AAA CGA CTA A-3 for occludin; 5-TCC GTG TTG TGG ATA CCT TGT A-3 and 5-GCC TCG TTC TAC CTC CTT ATG A-3 for ZO-1; 5-GAA GGG CTC ATG ACC ACA GTC CAT-3 and 5-TCA TTG TCG TAC CAG GAA ATG AGC TT-3 for GAPDH. GAPDH was chosen for housekeeping gene for normalizing the data of gene expression. Each experiment was repeated at least three times. The relative quantification of the gene was determined using the comparative CT method (2^−DDCt^).

### Western Blot Analysis

After different treatments, cells were lysed in ice-cold RIPA (Applygen Technologies Company, Beijing) containing protease inhibitor PMSF. Protein concentration was determined using the BCA protein assay kit (Eppendorf-Bio photometer, Germany). The western blot was performed as described in a previous study [21]. The following primary antibodies were used: claudin-5 (1∶400), occludin (1∶400), ZO-1(1∶400) (Invitrogen, Carlsbad, CA, USA) and β-actin (1∶1000, Abcam, Cambridge, MA, USA). The appropriate secondary anti-rabbit/mouse IgG antibodies (1∶10,000, Abcam, Cambridge, MA, USA) were used. β-actin was chosen for housekeeping gene for normalizing the data of protein expression. The bands were visualized by Western Lighting chemiluminiscence reagent (ProteinSimple, USA) and quantified by densitometry using Quantity One software.

### Immunofluorescence Assay

After treatment, immunofluorescence was performed as previously described [22]. HUVECs and bEnd.3 were incubated with fluorescein isothiocyanate (FITC)-conjugated primary antibodies (occludin, 1∶200; claudin-1/3/4/5/7, 1∶200; ZO-1, 1∶200) over night at 4°C. Then, cells were washed triple using wash buffer and incubated with dye for F-actin (Rhodamine Phalloidin, 1∶1000) for 1 hr at room temperature. DAPI (1∶1000) was used for staining cellular nuclear. The cells were washed for three times and observed under a fluorescence microscope (Laica, TCS SP5II, Germany).

### Paracellular Permeability Assay

Flux of FITC-conjugated dextran (FITC-dextran, 10 kDa, Sigma) across HUVECs and bEnd.3s monolayers was used to measure the paracellular permeability [23]. Briefly, HUVECs and bEnd.3s were seeded at a density of 2×10^4^ cells/well in 300 µL medium onto polycarbonate 24-well transwell chambers with a 0.4 µm mean pore size and a 0.3 cm^2^ surface area (Millicell Hanging Cell Culture Inserts, USA). Cells were incubated with FITC-dextran (1 mg/mL) in HBSS buffer for 90 min. Thereafter, relative fluorescence passed through the chamber (in the lower chambers) was determined by using EnSpire Manager (PerkinElmer Company, USA) multimode plate reader at an excitation wavelength of 485 nm and an emission wavelength of 535 nm.

Restriction of paracellular transport was determined by analyzing the apparent permeability coefficient (Papp) for FITC-dextran across the cells. Papp was calculated by the following equation 
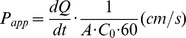



where dQ /dt is the amount of FITC transported per minute (ng/min), A is the surface area of the filter (cm^2^), C_0_ is the initial concentration of FITC(ng/ml) and 60 is the conversion from minutes to seconds.

We detected the permeability of HUVECs and bEnd.3s daily for continuous 8 or 11 days after cells seeded to determine the timing of EC barrier formation. In our experimental conditions, the time of EC barrier formation was day 4 and maintained for 4–6 days as reported by Horiuchi T et al and Koto T *et al* [24], [25]. Thus, we chose to expose HUVECs and bEnd.3s to hypoxia/OGD with/without citicoline on the fourth day after cells seeded, the data were obtained 24 h (for hypoxia) or 6 h (for OGD) after the different treatments.

### Statistical Analysis

Data were all expressed as the mean±SD. Comparisons for two groups were performed by using a Student's t-test (GraphPad Prism 5 software). *P*-values of <0.05 were considered to be significant.

## Results

### Citicoline Ameliorates Hypoxia/OGD-induced Increase in the Permeability of Endothelial Cells

**Figure 1:**
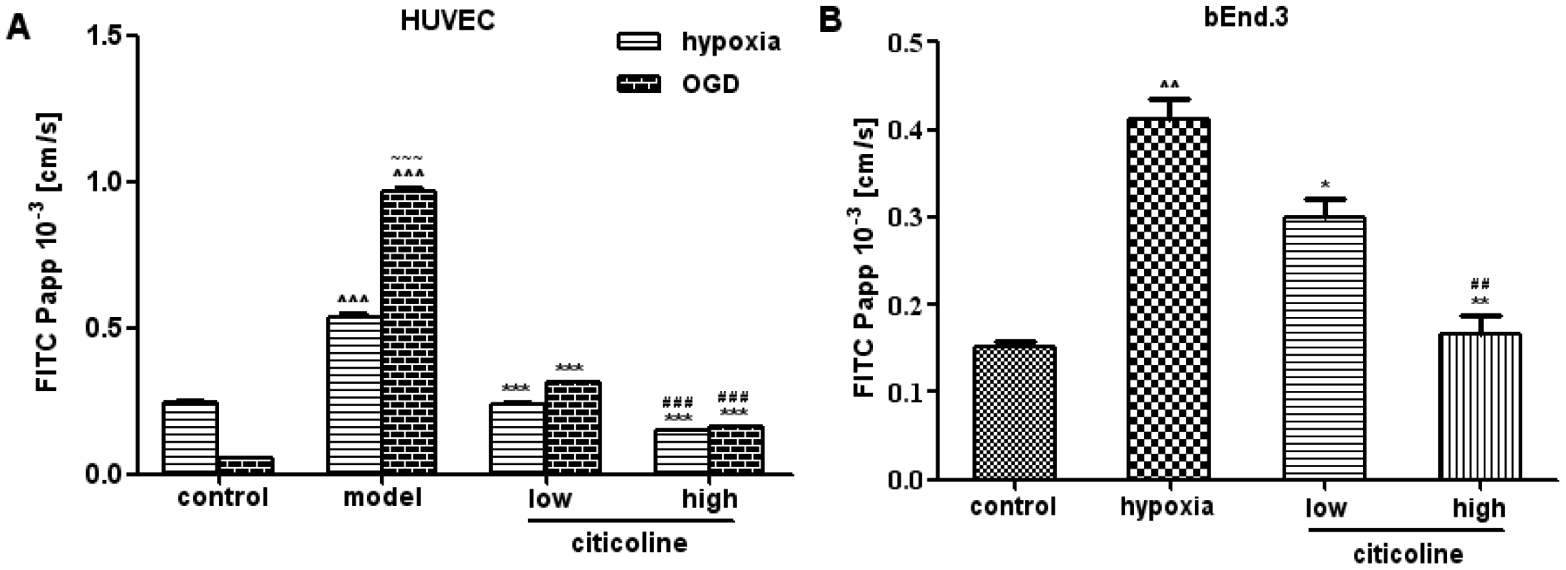
The effects of citicoline on Hypoxia/OGD induced increase of permeability in endothelial cells. Hypoxia/OGD induces the increase of permeability. Citicoline decreases the permeability in HUVECs (**A**) and bEnd.3s (**B**) under hypoxia/OGD. High-dose is more effective when compared to low dose. ^∧∧^ *P*<0.01, ^∧∧∧^ *P*<0.001 *vs.* control; *P<0.05, **P<0.01, ***P<0.001 *vs.* model; ^##^ *P*<0.01, ^###^ *P*<0.001 *vs.* low citicoline; ^∼∼∼^ *P*<0.001 *vs.* hypoxia, n = 3/group. Papp: apparent permeability coefficient.

As shown in Fig. 1A, the permeability increased dramatically after hypoxia (0.25±0.01×10^−3^ cm/s and 0.53±0.02×10^−3^ cm/s, control *vs.* hypoxia *P*<0.001) and OGD (0.08±0.001×10^−3^ cm/s and 0.99±0.04×10^−3^ cm/s, control *vs.* OGD *P*<0.001) in HUVECs. OGD induced more severe disruption in barrier function than hypoxia (*P*<0.001). Both low and high dose of citicoline decreased FITC permeability (*P*<0.001). High dose had more effectives than low dose (*P*<0.001).

As shown in Fig. 1B, the permeability increased dramatically after hypoxia (0.15±0.005×10^−3^ cm/s and 0.41±0.02×10^−3^ cm/s, control *vs.* hypoxia *P*<0.01) in bEnd.3s. Both low dose and high dose of citicoline decreased FITC permeability (*P*<0.05 or 0.01). High dose had more effectives than low dose (*P*<0.01).

### Citicoline Improves the Linear Distribution of TJPs in the Membrane of Endothelial Cells Disrupted by Hypoxia/OGD

**Figure 2:**
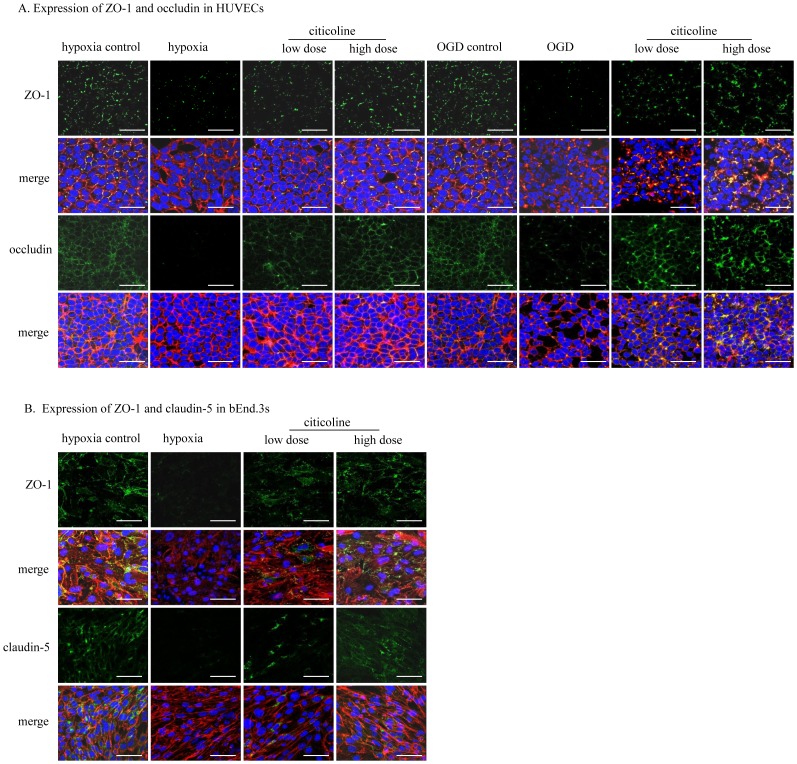
The effects of citicoline on Hypoxia/OGD-induced disruption of TJPs expression in endothelial cells. Representative images of TJP staining on endothelial cells. Green: ZO-1/occluding/claudin-5; Red: F-actin; Blue: nuclear. Scale bars: 50 µm. Hypoxia/OGD decreases the expression and results in a discontinuous distribution of ZO-1 and occludin on the membrane of HUVECs (**A**). Exposure to hypoxia causes a decreased expression and discontinuous distribution of ZO-1 and claudin-5 on the membrane of bEnd.3s (**B**). Treatment of citicoline restores the linear distribution of ZO-1, occludin and claudin-5.

In HUVECs under normoxia condition, cells exhibited continuous membranous staining of ZO-1 and occludin (Fig. 2A). While claudin-5 scarcely expressed on the membrane, and the trace of claudin-1,3,4,7 failed to be found (data not shown). After hypoxia or OGD, HUVEC showed a discontinuous staining of ZO-1 and occludin (Fig. 2A). Disrupted expression of TJPs under hypoxia or OGD conditions corresponded to an increased paracellular permeability for FITC-dextran which determined above. Interestingly, citicoline treatment restored the linear distribution of ZO-1 and occludin (Fig. 2A).

In normoxia condition, bEnd.3s showed well-defined membrane staining signal of ZO-1 and claudin-5 (Fig. 2B). Exposure to hypoxia resulted in a loss of the continuous staining pattern of both ZO-1 and claudin-5. After citicoline treatment, bEnd.3s showed continuous staining of ZO-1 and claudin-5 (Fig. 2B).

### Citicoline Increases the Expression of TJPs at Both mRNA and Protein Levels in Endothelial Cells

**Figure 3:**
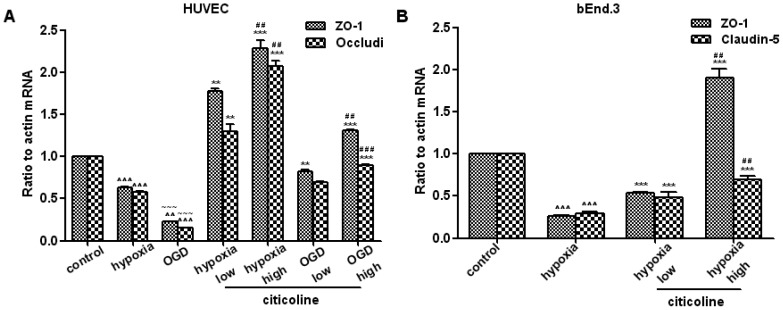
The effects of citicoline on Hypoxia/OGD induced changes in the mRNA level of TJPs in endothelial cells. (**A**) mRNA level of ZO-1 and occludin in HUVECs. (**B**) mRNA levels of claudin-5 and ZO-1 in bEnd.3s. Both high and low dose of citicoline increase the expression, while high-dose is more effective when compared to low dose. ^∧∧^ *P*<0.01, ^∧∧∧^ *P*<0.001 *vs.* control; *P<0.05, **P<0.01, ***P<0.001 *vs.* model; ^##^ *P*<0.01, ^###^ *P*<0.001 *vs.* low citicoline; ^∼∼∼^ *P*<0.001 *vs.* hypoxia, n = 3/group.

**Figure 4:**
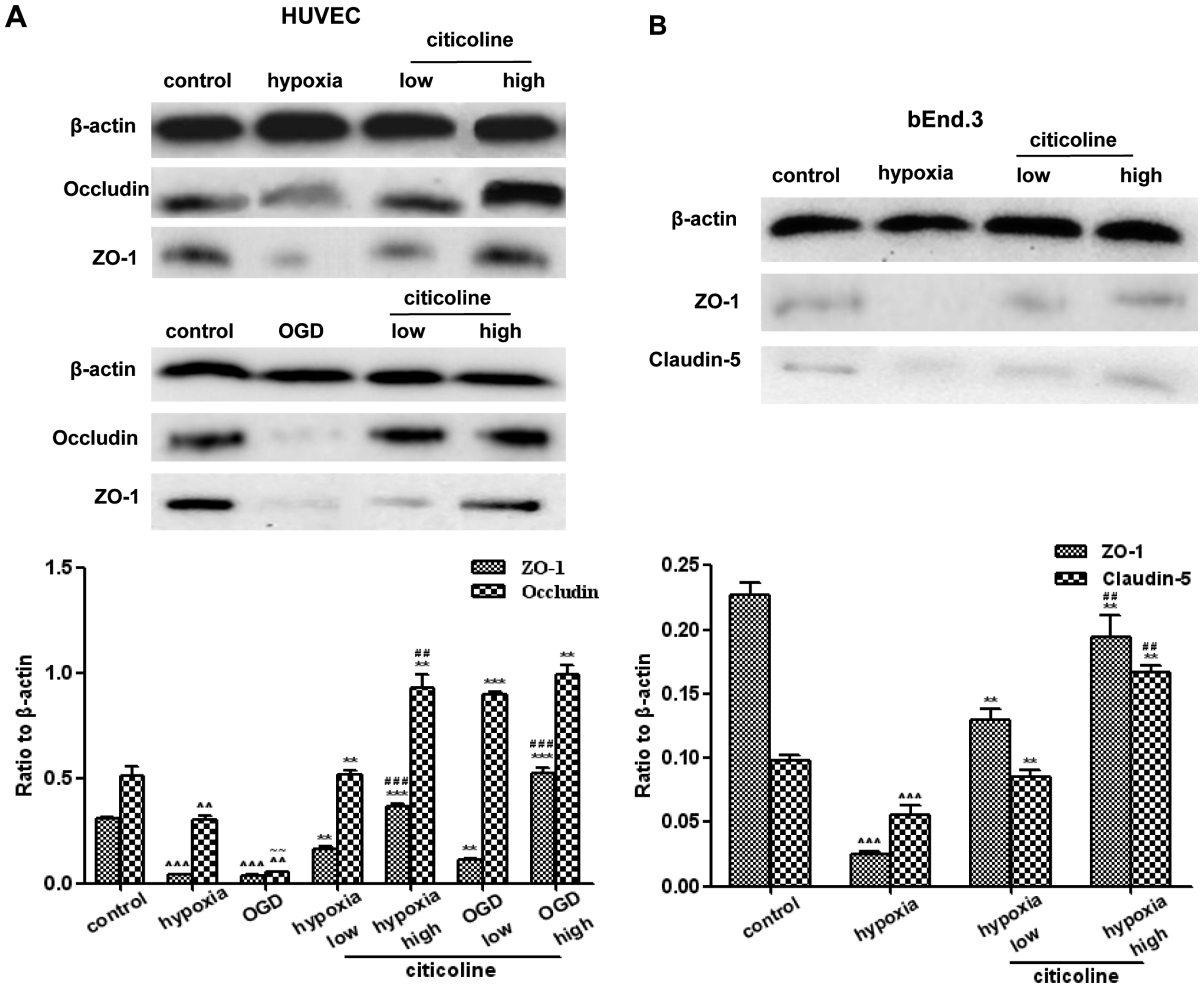
The effects of citicoline on Hypoxia/OGD induced changes in the protein expression of TJPs in endothelial cells. (**A**) Protein levels of ZO-1and occludin in HUVECs. (**B**) Protein levels of ZO-1 and claudin-5 in bEnd.3s. Both high and low dose of citicoline increase the expression, while high-dose is more effective when compared to low dose. ^∧∧^ *P*<0.01, ^∧∧∧^ *P*<0.001 *vs.* control; *P<0.05, **P<0.01, ***P<0.001 *vs.* model; ^##^ *P*<0.01, ^###^ *P*<0.001 *vs.* low citicoline; ^∼∼^ *P*<0.01 *vs.* hypoxia, n = 3/group.

As shown in Fig. 3A and 4A, both hypoxia and OGD induced significant decrease on the expressions of ZO-1 and occludin (at mRNA and protein levels) in HUVECs (*P*<0.01). OGD caused more obvious changes when compared to hypoxia (ZO-1 at mRNA level, occludin at mRNA and protein levels). Both high-dose and low-dose of citicoline increased the expression of ZO-1 and occludin under hypoxia or OGD (*P*<0.01), while the high-dose of citicoline had more efficiency in up-regulating the expression.

In bEnd.3s, hypoxia induced dramatically decrease of ZO-1 and claudin-5 in both mRNA and protein levels (*P*<0.01). After citicoline treatment, the expression of ZO-1 and claudin-5 were increased (*P*<0.01), and high-dose of citicoline had more efficiency (Fig. 3B and 4B).

## Discussion

In our present study, we demonstrate for the first time that citicoline improves the endothelial barrier function impaired by hypoxia/OGD via upregulating the expression of TJPs.

Hypoxia or OGD has been demonstrated to cause endothelial cell barrier dysfunction [18], [25], [26]. HUVECs and bEnd.3s are suitable cells for studying endothelial barrier function because of their defined TJPs and adheren junction characteristics [22], [27]. Thus, we used hypoxia and OGD conditions to establish *in vitro* endothelial barrier breakdown models in these two endothelial cell lines. There are experiments reporting that 24 h/6 h OGD or 24 h hypoxia destroys endothelial barrier function [18], [25]. We tested these conditions in our pilot study and found out that 24 h OGD caused significant cell death while 6 h OGD did not (data not shown). Thus, for avoiding the influence of cell death on functional study, we chose 24 h hypoxia and 6 h OGD to build endothelial barrier disruption models. Consistently with previous studies, our results showed that hypoxia/OGD induces the increase of paracellular permeability in HUVECs and bEnd.3s.

Citicoline has been widely accepted to be effective for treating neurodegenerative diseases, such as PD and AD [1]. Recent animal experiments suggest its therapeutic effects on ischemic stroke [28]. In the present study, we reveal that citicoline dose-dependently ameliorates the endothelial barrier dysfunction in HUVECs and bEnd.3s. This is in agreement with a previous study reporting that citicoline reduces ischemia-induced brain edema in gerbils [29]. And also it is consistent with a clinical research showing that the acute ischemic stroke patients who received high dose of citicoline get better neurological and functional outcomes than those who received the low dose [30]. Since endothelial cells play an important role in the barrier function, our data suggests that citicoline could be an effective therapeutic drug for treating diseases characterized by endothelial barrier disruption.

Furthermore, the molecular basis of citicoline in improving endothelial cell barrier function was investigated. The expression of TJPs has been reported to contribute to barrier function [14], [15]. Tight junction is constituted by different kinds of TJPs such as claudin family, junctional adhesion molecules and ZO family. ZO-1 and claudin-5 are the most important components for cell barrier integrity. Claudin-5 can greatly reduce dextran permeability and improve transendothelial electrical resistance [31], and plays an essential role in the earliest stage of CNS angiogenesis [32]. ZO-1 serves as a bridge between transmembrane proteins and skeleton proteins, and this interaction is important to the stability and function of endothelial barrier [33], [34]. Occludin has also been suggested to play a key role in the barrier function of the TJPs [35]. Therefore, we focused on these TJPs in this study. We found that hypoxia/OGD resulted in claudin-5, occludin and ZO-1 down-regulation and their linear distribution on the membrane of endothelial cells. Citicoline treatment was effective in restoring their expression and linear distribution in a dose-dependent manner. Our data reveal a novel pharmacological effect of citicoline on endothelial barrier, which could offer a new approach for treating ischemic diseases, although the results from clinical trials are controversy [36]. The mechanism by which citicoline regulates TJPs was not explored in this study and deserves further investigation. Finally, we recognize the potential role of inflammatory cytokines in mediating EC barrier dysfunction. We will include this consideration in our future study in animal models.

Overall, the present results demonstrate that citicoline could restore the endothelial barrier function compromised by hypoxia/OGD through its ability to upregulate the expression of TJPs including ZO-1, occludin and claudin-5. Further *in vivo* studies are needed for the proof of conception.
